# Dysregulated expression of cell surface glycoprotein CDCP1 in prostate cancer

**DOI:** 10.18632/oncotarget.6193

**Published:** 2015-10-20

**Authors:** Lifang Yang, Sucharita M. Dutta, Dean A. Troyer, Jefferson B. Lin, Raymond A. Lance, Julius O. Nyalwidhe, Richard R Drake, O. John Semmes

**Affiliations:** ^1^ Leroy T. Canoles Jr. Cancer Research Center, Eastern Virginia Medical School, Norfolk, VA, USA; ^2^ Department of Microbiology and Molecular Cell Biology, Eastern Virginia Medical School, Norfolk, VA, USA; ^3^ Department of Pathology and Anatomy, Eastern Virginia Medical School, Norfolk, VA, USA; ^4^ Urology of Virginia, Norfolk, VA, USA; ^5^ Department of Cell and Molecular Pharmacology and Experimental Therapeutics, Medical University of South Carolina, Charleston, SC, USA

**Keywords:** glycoprotein, CUB-domain containing protein 1, prostate cancer, biomarker

## Abstract

CUB-domain-containing protein 1 (CDCP1) is a trans-membrane protein regulator of cell adhesion with a potent pro-migratory function in tumors. Given that proteolytic cleavage of the ectodomain correlates with outside-in oncogenic signaling, we characterized glycosylation in the context of cellular processing and expression of CDCP1 in prostate cancer. We detected 135 kDa full-length and proteolytic processed 70 kDa species in a panel of PCa cell models. The relative expression of full-length CDCP1 correlated with the metastatic potential of syngeneic cell models and an increase in surface membrane expression of CDCP1 was observed in tumor compared to adjacent normal prostate tissues. We demonstrated that glycosylation of CDCP1 is a prerequisite for protein stability and plasma membrane localization, and that the expression level and extent of N-glycosylation of CDCP1 correlated with metastatic status. Interestingly, complex N-linked glycans with sialic acid chains were restricted to the N-terminal half of the ectodomain and absent in the truncated species. Characterization of the extracellular expression of CDCP1 identified novel circulating forms and revealed that extracellular vesicles provide additional processing pathways. Employing immunoaffinity mass spectrometry, we detected elevated levels of circulating CDCP1 in patient urine with high-risk disease. Our results establish that differential glycosylation, cell surface presentation and extracellular expression of CDCP1 are hallmarks of PCa progression.

## INTRODUCTION

Protein glycosylation is one of the most common and versatile post-translational modifications with a clear role in the regulation of numerous protein functions [1]. Altered glycosylation of cell surface proteins has been associated with cellular transformation and cancer progression underscoring a potential role in disease initiation and regulation [2–6]. We recently described a targeted approach for cell surface glycoprotein analysis and observed that CUB-domain-containing protein 1 (CDCP1) was over-expressed in metastatic prostate cancer [7].

CDCP1 is a type I single transmembrane protein also known as subtractive immunization associated 135 kDa (SIMA135) [8], gp140 [9], transmembrane and associated with Src kinases (Trask) [10], and CD318 [11]. CDCP1 is expressed by stem or progenitor cells in hematopoietic, mesenchymal and neural tissues [12, 13] and is overexpressed in solid tumors including breast [14], colon [15], kidney [16], pancreatic [17], and lung [18]. Experimental data support a role for CDCP1 in cancer progression [19, 20], ECM degradation [21], anchorage-independent signaling [22], and cancer cell resistance to anoikis [19]. In PCa, the potential of CDCP1 as a therapeutic target has been reported [23, 24], but its expression and potential clinical significance have not been fully analyzed.

The existence of 14 consensus N-glycosylation motifs and 3 proteolytic cleavage sites in the extracellular domain together with 5 tyrosine residues in the intracellular C-terminus reflects the functional role for CDCP1 in outside-in signal transduction. In fact, proteolytic cleavage of full-length CDCP1 results in a smaller “activated” protein, which is a substrate for Src-mediated tyrosine phosphorylation and a scaffold for the recruitment of PKCδ [9, 10, 25]. The two membrane-bound forms of CDCP1, the HMW-CDCP1 and a shorter LMW-CDCP1 species, have been observed in various cancer cells [9, 10, 19–21, 26] and keratinocytes [27]. The LMW-CDCP1 is generated from HMW-CDCP1 through the action of exogenous serine proteases via cleavage in the ectodomain at R368 and K369 [9, 10, 25]. In cancer cells, both forms of CDCP1 are tyrosine phosphorylated in the event of cell adhesion [22]. Although CDCP1 phosphorylation and proteolysis are well documented [28–32], the glycosylation status and its role in CDCP1 biology are not known.

In the present study, we characterized the glycosylation of CDCP1 in prostate derived cell lines using glycan-specific enzymes and glycosylation inhibitors. In addition, we evaluated the differential glycosylation and expression of CDCP1 between aggressive and non-aggressive prostate cancer cells and human tissues. We show that glycosylation determines protein stability, ectodomain processing and extracellular expression of CDCP1. We identified novel circulating extracellular forms of CDCP1 that displayed disease-specific expression in patient urine. Our findings suggest that glycosylation regulates the expression of extracellular forms of CDCP1 that correlate to disease state.

## RESULTS

### Differential expression of CDCP1 in human prostate cancer cells

**Figure 1:**
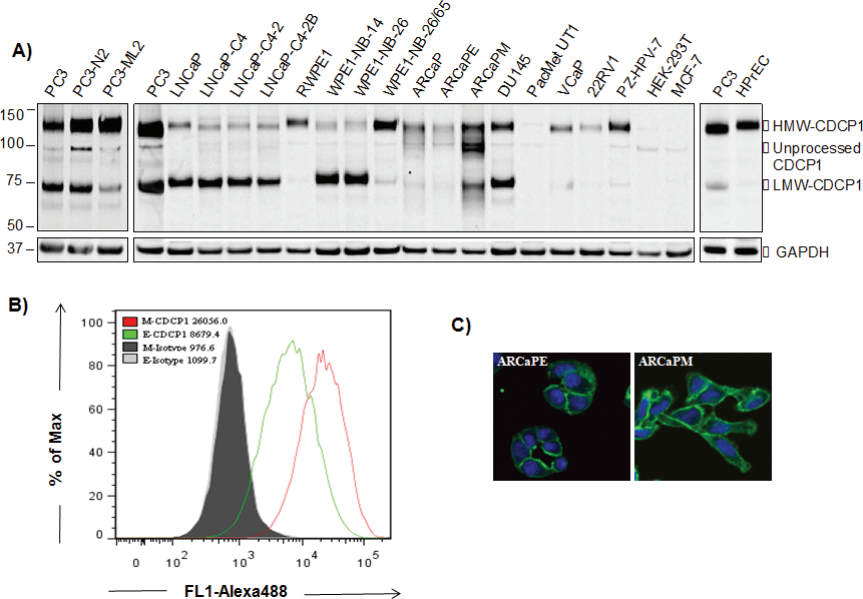
Expression of CDCP1 in human prostatic cell lines **(A)** Expression of CDCP1 in human prostatic cell lines. Whole cell lysates were subjected to western blot analysis using anti-CDCP1 (CS4115). Extracts were normalized to total protein and expression of GAPDH used as a loading control. HEK-293T and MCF-7 cells served as negative controls. Surface expression of CDCP1 in ARCaP EMT model. Cell surface CDCP1 expression was detected by flow cytometric analysis **(B)** and immunofluorescence microscopy **(C)** using anti-CDCP1 (AF2666). The nucleus was counterstained with TO-PRO3 (blue). In flow cytometric analysis, living cells, which are PI negative, were gated for analysis. The relative expression of CDCP1 in ARCaPE and ARCaPM cells was calculated by mean fluorescence intensity.

Previous experimental data for CDCP1 in prostate cancer has been restricted to the study of PC3 and DU145 cancer models. In order to derive a broader consensus, we surveyed a panel of prostate cell lines for expression of CDCP1. As shown in Figure 1A, CDCP1 protein was detected in 19 of 20 prostate cell lines displaying variable expression of full-length 135 kDa (HMW-CDCP1) and truncated 70 kDa (LMW-CDCP1). PC3 lines displayed the highest staining for CDCP1 while PacMetUT1 showed only marginal expression. The normal prostatic cell line HPrEC, and immortalized RWPE-1 and PZ-HPV-7, expressed predominately HMW-CDCP1. In the PCa syngeneic models, the metastatic sublines displayed increased HMW-CDCP1 compared to the low metastatic counterparts (PC3-N2 vs PC3-ML2, WPE-NB-26/65 vs RWPE-1, and ARCaPM vs ARCaPE). LNCaP cell lines with differing androgen responsiveness, showed static expression of CDCP1 with higher levels of LMW-CDCP1.

We extended the analysis of the ARCaP epithelial to mesenchymal transition (EMT) model to examine differential surface expression of CDCP1 related to the transition phenotype. Employing FACS analysis, we observed increased surface expression of CDCP1 in the mesenchymal-like ARCaPM compared to the epithelial-like ARCaPE cells (Figure 1B). This finding was visually confirmed with confocal microscopy showing that surface expression of CDCP1 was higher in ARCaPM (Figure 1C). These results suggest an association of CDCP1 surface expression with the metastatic potential of PCa.

### CDCP1 expression in prostate cancer tissues

**Figure 2:**
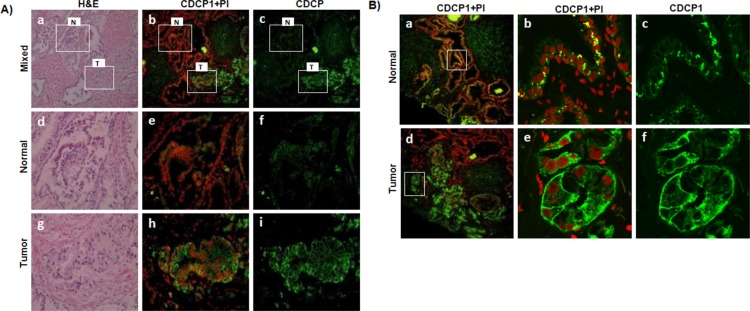
Expression of CDCP1 in prostate patient tissues **(A)** Immunofluorescence microscopy of CDCP1 expression in matched normal (N) and cancerous (T) frozen prostate tissues from a prostate cancer patient (Gleason score = 4+3). Frozen tissue sections (6 μm) were stained with H&E and a serial section analyzed with anti-CDCP1 (mAb41–2, green). Nuclei were counterstained with propidium iodide (red). Shown (panels a-c) are a lower magnification view (100X) of a region of tumor (T) and adjacent normal (N). High magnification (400X) of selected regions from normal (panels d-f) and tumor (panels g-i) glands correspond to the indicated areas marked in the top panels (white box). **(B)** Immunofluorescence analysis of CDCP1 subcellular localization. Frozen PCa sections were stained with anti-CDCP1 (mAb41–2, green) and the nuclei counterstained with propidium iodide (red). Representative images for normal (a-c) and tumor (d-e) glands are shown. High magnification of selected regions from normal (b, c) and tumor (e, f) glands as indicated in the left panels (white box). Magnification 100X (a, d), 880X (b, c), 1040X (e, f).

We examined the expression of CDCP1 in frozen human prostate cancer tissues via immunofluorescence microscopy (Figure 2). Although CDCP1 was observed in both normal prostate epithelial and malignant cells, the staining intensity and subcellular localization were disparate. Human prostate cancer cells had a focally higher reactivity when compared to adjacent normal (Figure 2A, panels a-c). Malignant cells expressing CDCP1 protein belonged to a single acinus or to a few adjacent acini when compared to adjacent normal (Figure 2A, compare panels d-f to panels g-i). The tumor staining was predominantly localized to the plasma membrane (Figure 2B, d-f). The basal and apical surface demonstrated more concentrated CDCP1 than lateral membranes. In contrast, adjacent normal cells expressed CDCP1 in discrete juxtanuclear compartments, close to the basal side (Figure 2B, a-c) with dramatically reduced plasma membrane expression (Figure 2B, compare c to f). The proportion of focally positive regions varied across malignant glands but was absent in all normal glands examined.

We examined a tissue microarray containing 100 human primary prostate cancer specimens for expression of CDCP1 in tumors compared with adjacent normal epithelial tissue (Supplemental Table S1 and Figure S1). The pronounced differential localization of CDCP1 observed in frozen sections was not observed in FFPE tissues. We observed variation in the intensity of CDCP1 expression and a reduction in the median staining intensity in tumor regions, consistent with an earlier report [24]. Other cell types including blood, endothelium, vascular smooth muscle, and prostatic stroma fibroblasts, were negative. In summary, although localization of CDCP1 is altered, the overall expression in prostate cancer tissues as performed on FFPE material is heterogeneous making CDCP1 a poor candidate as a tissue based biomarker.

### Role of N-glycosylation on CDCP1 plasma membrane localization

**Figure 3:**
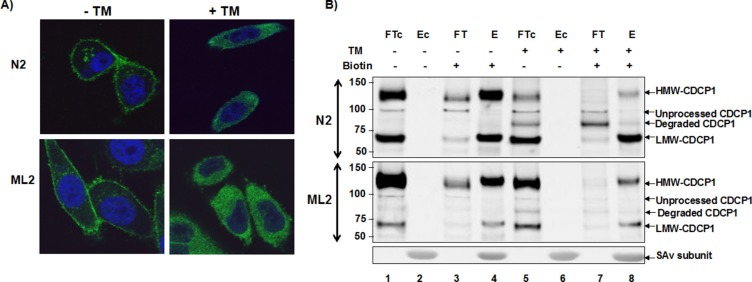
Surface expression of CDCP1 requires N-glycosylation **(A)** PC3-N2 and PC3-ML2 cells were either treated with 1 μg/ml tunicamycin for 24 h (+TM) or untreated (−TM). The cells were then fixed and subjected to immunofluorescence confocal microscopy using anti-CDCP1 antibody (CS4115, green). The nucleus was counterstained with TO-PRO3 (blue) and the images merged. **(B)** Protein extracts were prepared from PC3-N2 and ML2 cells that had been surface biotinylated (+) or untreated (−) and additionally grown in the presence (+) or absence (−) of tunicamycin. Biotinylated and nonbiotinylated protein fractions were separated by streptavidin affinity. The biotinylated bound (E) and unbound (FT) fractions, non-biotinylated bound (Ec) and unbound (FTc) fractions were separated by SDS-PAGE and immunoblotted with ant-CDCP1 (CS4115).

To establish the role of glycosylation in surface expression, we examined the sub-cellular localization of CDCP1 following inhibition of N-glycosylation. The PC3 sublines, PC3-N2 (low metastatic potential) and PC3-ML2 (high metastatic potential) were grown under subconfluent conditions in the presence (+TM) or absence (−TM) tunicamycin. In figure 3A we show strong cellular immunostaining for CDCP1 on both PC3 subtypes. However, treatment with TM resulted in significant changes in staining pattern with increased intensity in the cytoplasm.

The dependence of CDCP1 surface expression on N-glycosylation was orthogonally confirmed via cell surface biotinylation. This approach preferentially labels the exposed primary amines of proteins on the surface of cells and exploits the strong interaction between biotin and streptavidin for the purification of cell-surface proteins. The purified surface proteins were separated by SDS-PAGE and probed for expression of CDCP1. As shown in Figure 3B, the majority of HMW-CDCP1 and LMW-CDCP1 was found in the cell surface biotinylated fraction (E) as opposed to the surface inaccessible fraction (FT). Upon TM treatment, the surface expression of HMW-CDCP1 decreased concomitant with the appearance of an 80 kDa protein. The observance of TM-dependent cytoplasmic expression of a degraded protein suggests an important role for N-glycosylation in CDCP1 stability and turnover. In contrast, LMW-CDCP1 was resistant to TM treatment. These results support previous observations that LMW-CDCP1 derives from proteolysis of HMW-CDCP1 and is subject to slower turnover [25].

We also noted a differential sensitivity of HMW-CDCP1 to TM treatment between N2 and ML2 cell lines. This was observed by comparing the TM-induced reduction inintensity of HMW-CDCP1 from the cell lysates (Figure 3B, lanes 1 and 5) and isolated surface sialoglycoproteins (Figure 3B, lanes 4 and 8). In fact, HMW-CDCP1 from N2 cells displayed an ID_50_ of about 0.05 ug/ml TM and a half-life of 3 hours (see Supplemental Figure S2). In contrast, HMW-CDCP1 in the ML2 cells displayed an approximately 4-fold higher ID_50_ and 3-fold increased half-life. These data demonstrate a structural difference in HMW-CDCP1 in N2 versus ML2 cell lines.

### Characterization of CDCP1 glycosylation

**Figure 4:**
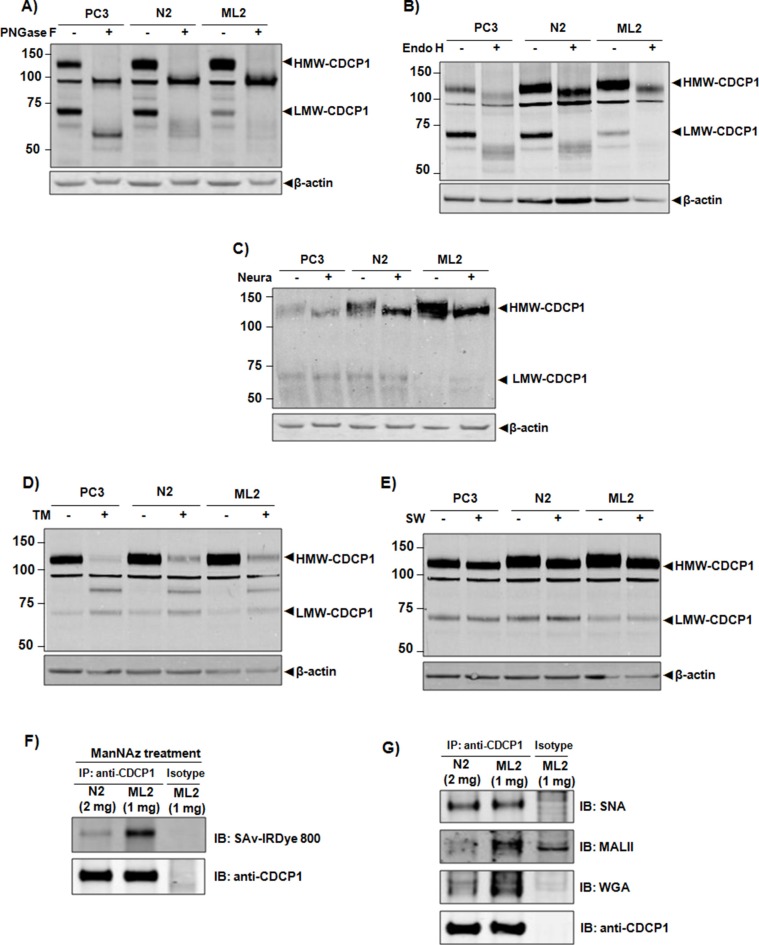
Characterization of CDCP1 glycosylation *In vitro* deglycosylation of CDCP1 employing Neuraminidase **(A)** Endo H **(B)** and PNGase F **(C)**. Hydrolyzed lysates from PC3, N2, and ML2 cells were separated on SDS-PAGE and immunoblotted with anti-CDCP1 (CS4115). *In vivo* inhibition of glycosylation of CDCP1 in which PC3, N2, and ML2 cells were treated with tunicamycin **(D)** or swainsonine **(E)** in vivo for 24 h. The total cell lysate was extracted, subjected to SDS-PAGE and immunoblotted with anti-CDCP1 (CS4115). β-actin was used as a loading control. Shown are HMW-CDCP1 and LMW-CDCP1. **(F)** Sialylation of HMW-CDCP1 protein was quantified by metabolically labeling sialyl proteins with ManNAz followed by immunoprecipitation of normalized amounts of CDCP1 with anti-CDCP1 (CS4115). A click reaction was performed to label the azido-sugar with biotin to allow for subsequent blotting with IRDye 800-conjugated streptavidin. **(G)** Normalized amounts of HMW-CDCP1 from N2 and ML2 cell was immunoprecipitated with anti-CDCP1 (CS4115) subjected to SDS-PAGE and immunoblotted with linkage-specific lectins SNA, MALII, and WGA as indicated.

Though known to be extensively glycosylated, the carbohydrate structure of CDCP1 has not been reported. We employed neuraminidase (hydrolyzes terminal sialic acid residues), endoglycosidase H (Endo H, preferentially cleaves high mannose and hybrid oligosaccharides structures), and peptide-N-glycosidase F (PNGase F, cleaves asparagine-linked (N-linked) oligosaccharides) to determine the relative sensitivity of CDCP1 as a substrate for each enzyme. Treatment with PNGaseF produced a 90 kDa and 53 kDa band consistent with the predicted apparent mass of completely deglycosylated full-length and truncated CDCP1 (Figure 4A). Digestion with Endo H led to a mass shift of approximately 15 kDa for HMW-CDCP1 (Figure 4B), indicating that a portion of the CDCP1 glycan is resistant. In contrast, Endo H treatment of LMW-CDCP1 produced several broad bands at or near the apparent molecular weight of completely deglycosylated LMW-CDCP1 (Figure 4, compare panels 4A and 4B). These results demonstrate that high-mannose or hybrid oligosaccharide chains contribute to a proportion of N-glycans present on CDCP1. Treatment with neuraminidase resulted in a decrease in HMW-CDCP1, whereas there was no detectable change in the LMW band (Figure 4C), indicating a higher sialic acid content in N-glycans of HMW-CDCP1 compared to LMW-CDCP1.

To assess the glycosylation status of CDCP1 *in vivo*, PC3 cells were treated with the N-linked glycosylation inhibitor tunicamycin (TM) or the mannosidase II inhibitor swainsonine (SW). Exposure to TM resulted in production of an 80 kDa species that is smaller than completely deglycosylated CDCP1 (Figure 4D), suggesting that inhibition of glycosylation results in protein degradation. By comparison, LMW-CDCP1 was resistant to TM treatment. Treatment with SW decreased the mass of HMW-CDCP1, but had no obvious effect on LMW-CDCP1 (Figure 4E). This result suggests the addition of complex-type N-glycans to HMW-CDCP1 but not LMW-CDCP1, consistent with results obtained from treatment with Endo H. Taken together, these data suggest that the carbohydrate structures present on CDCP1 are high-mannose/hybrid-type N-linked glycans with complex N-glycans terminated by sialic acid residues clustered in the N-terminal ectodomain region.

Having determined that sialylation is predominately confined to the ectodomain, we examined the sialylationstatus of HMW-CDCP1. PC3 cells were incubated with ManNAz to label sialic acid containing glycoproteins. As shown in Figure 4F, equal amounts of HMW-CDCP1 were immunoprecipitated from the PC3 sublines (although two-fold excess N2 to ML2 lysate was required). When probed for sialyl glycans using labeled streptavidin conjugate, the relative sialylation of HMW-CDCP1 was notably higher in the ML2 subline. To determine structural differences in sialylation, normalized amounts of CDCP1 were immunoprecipitated from cell lysates and subjected to lectin blotting using *Sambucus nigra lectin* (SNA, binds α2,6-linked sialic acid), *Maackia amurensis* lectin II (MALII, binds α2,3-linked sialic acid) or Wheat germ agglutinin (WGA, binds polysialic acid). The lectin affinity analysis indicated that sialylation via α2,6 linkage was observed in HMW-CDCP1 from both cells but the presence of α2,3 linkages and polysialic acid structures were preferentially expressed in HMW-CDCP1 of the ML2 subtype (Figure 4G). These results support that higher-order sialylation of CDCP1 is correlated with a metastatic phenotype in prostate cancer.

### Expression of extracellular CDCP1

**Figure 5:**
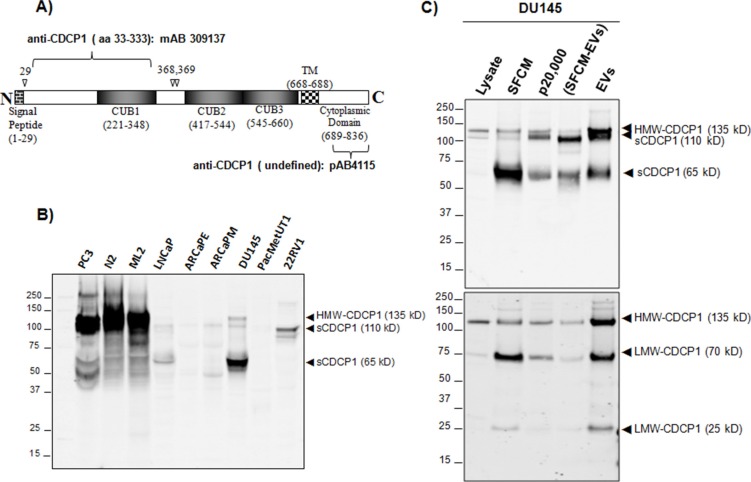
Analysis of extracellular forms of CDCP1 **(A)** A graphic representation of CDCP1 with important structural features noted. Shown is the cleavage site for processing of the membrane signal peptide (aa29) and extracellular processing of the ectodomain (aa368, 369). Antibodies targeting the extracellular domain and intracellular domain are indicated juxtaposed to the CDCP1 epitope. **(B)** Western analysis of indicated prostate cell lines with anti-CDCP1 (mAB309137) that only recognizes the extracellular ectodomain. HMW-CDCP1 and soluble forms of CDCP1 are indicated. **(C)** Analysis of extracellular CDCP1 derived from DU145 prostate line. Whole cell lysates, serum-free conditioned medium (SFCM), supernatant following 20,000 g, SFCM depleted of extracellular vesicles (SFCM-EVs) and extracellular vesicle (EVs) fractions were analyzed as indicated. The same transfer filter was immunoblotted with anti-CDCP1 specific to the extracellular domain (mAB309137, upper panel) and to the intracellular domain (CS4115, lower panel).

Cleavage of the HMW-CDCP1 at amino acid 368 results in the membrane-bound 70 kDa LMW-CDCP1 and a 65 kDa soluble form [25]. CDCP1 is also present in extracellular vesicles isolated from prostate cancer cell lines [23]. Thus, we examined the extracellular expression of CDCP1 as soluble and vesicle bound protein. We employed antibodies specific for either the extracellular or intracellular regions of CDCP1 (Figure 5A). The ectodomain specific antibody was raised against amino acids 33 to 333 and recognizes the 135 kDa HMW-CDCP1 and the soluble 65 kDa protein but not the 70 kDa LMW-CDCP1. The intracellular specific antibody will recognize membrane-bound HMW-CDCP1 and LMW-CDCP1 but not soluble extracellular forms of CDCP1 cleaved from the membrane. When we examined serum-free condition medium (SFCM) for expression of CDCP1 using the ectodomain specific antibody we observed the HMW 135 kDa species in PC3 and DU145 lines (Figure 5B). Interestingly, we observed 110 kDa band in LNCaP, ARCaPE, ARCaPM and 22RV1. Analysis of DU145, the cell line in which the soluble 65 kDa form was first described, yielded a prominent 65 kDa band, HMW-CDCP1 and the novel 110 kDa species. Note that the 65 kDa species observed in DU145 was not the 70 kDa LMW species since the extracellular domain specific antibody will not recognize that protein.

We expanded our analysis of CDCP1 in DU145 to include fractionation of whole cell lysate, SFCM, extracellular vesicle cleared SFCM (SFCM-EVs), and extracellular vesicles (EVs). We then separated the proteins via SDS-PAGE and immunoblotted with extracellular-specific (upper panel) and intracellular-specific (lower panel) CDCP1 antibodies (Figure 5C). The DU145 whole cell lysate revealed a HMW 135 kDa species that was recognized by both antibodies, as expected. In addition, whole cell lysate expressed the 70 kDa LMW-CDCP1 only recognized by the intracellular-specific antibody. Analysis of the SFCM revealed expression of the soluble 65 kDa CDCP1 as well as the 135 kDa HMW and 70 kDa LMW forms. This is reflective of the SFCM containing vesicle-bound forms of CDCP1. When we examined the extracellular vesicular fraction, we observed an enrichment of the 135 kDa HMW and 70 kDa LMW forms as would be expected if the membrane bound forms of CDCP1 were processed into extracellular vesicles. We also observed an enrichment of the soluble 65 kDa form in extracellular vesicles. Interestingly, we observed that the extracellular vesicular fraction was enriched for the novel 110 kDa ectodomain form as well as a 25 kDa membrane-bound cytoplasmic form of CDCP1. Cleavage of the HMW 135 kDa protein at the extracellular interface would produce a 110 kDa ectodomain and a 25 kDa membrane bound protein. These data demonstrate that CDCP1 processing and activation are linked to microvesicle formation.

**Figure 6:**
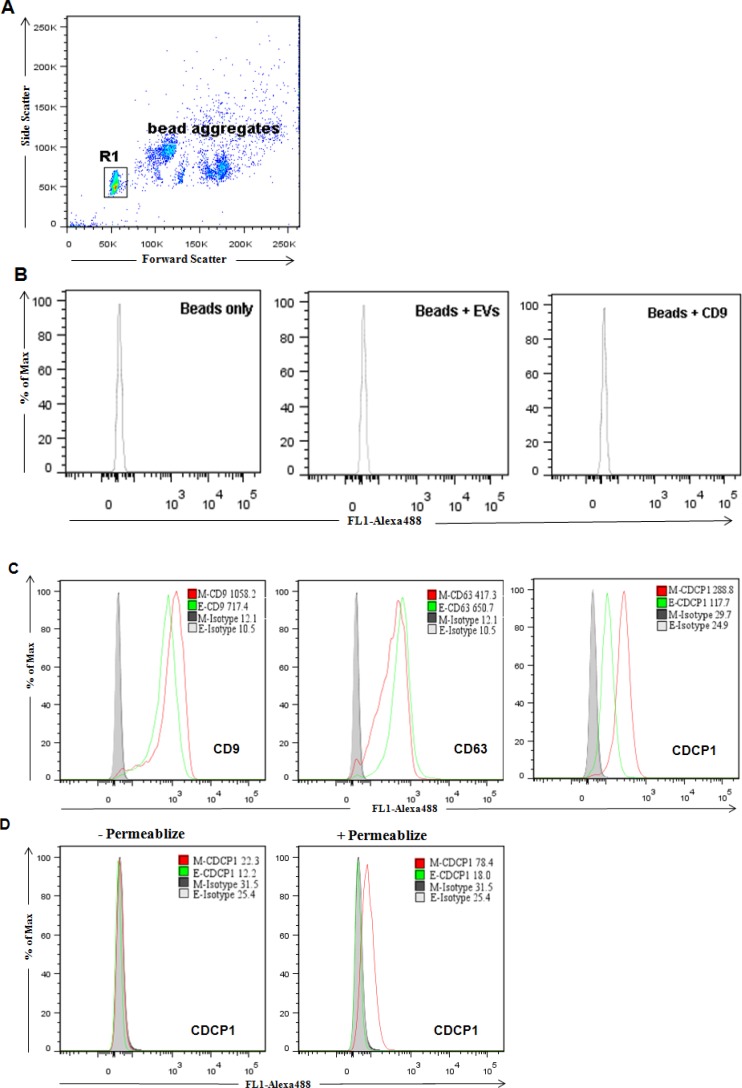
Flow cytometric analysis of surface protein expression on purified extracellular vesicles EVs purified from cell cultures were coupled to the surface of latex beads and analyzed for the presence of CD9, CD63 and CDCP1. **(A)** Only bead singlets (R1) typically representing 70–80% of total beads were gated. **(B)** Analysis of latex beads alone, with EVs or with CD9 were used as controls to evaluate nonspecific binding. **(C)** Detection of surface expression of CD9, CD63 and CDCP1 by antibodies targeting to extracellular domains. The relative expression of ARCaPE (green) and ARCaPM (red) are shown with isotype control (shaded peak). **(D)** Detection of expression of CDCP1 by an antibody (CS4115) that targets the intracellular C-terminus. EVs were analyzed with and without permeabilization, as indicated.

We employed FACS analysis to determine the membrane orientation of CDCP1 residing in extracellular vesicles. We bound microvesicles with latex beads to achieve a sufficient size for analysis on standard FACS instrumentation. A baseline performance was established by analysis of beads, beads plus microvesicles, and beads plus CD9 antibody (Figure 6A and 6B). When we examined extracellular vesicles isolated from ARCaP cells for expression of the exosome markers, CD9 and CD63, we observed specific expression and only marginal differences between ARCaPE and ARCaPM. However, the expression of CDCP1 on extracellular vesicles derived from ARCaPM was over two-fold greater than that of ARCaPE (Figure 6C). Similar results were achieved with western blot analysis of the same sample (Supplemental Figure S3), indicating the expression of extracellular forms of CDCP1 is correlated to the metastatic potentials of PCa cells. We then assessed extracellular vesicles for CDCP1 expression using an antibody specific for the intracellular domain of CDCP1 (Figure 6D). Expression was only detected following membrane permeabilization and was most prominent in the ARCaPM cells. These results establish that CDCP1 in extracellular vesicles is expressed as a type I transmembrane protein and retains the differential phenotype observed for the corresponding “producer” cells.

### Extracellular CDCP1 is overexpressed in men with high-risk prostate cancer

**Figure 7:**
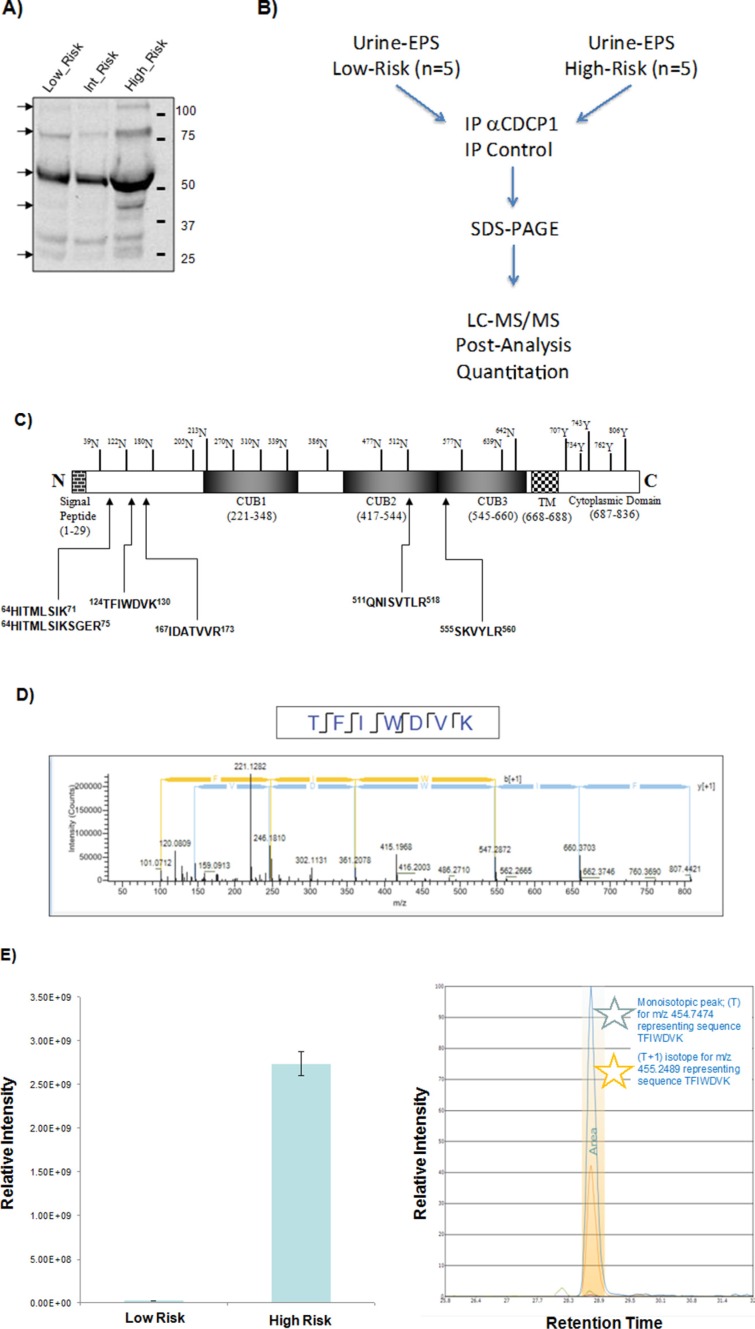
Quantitation of extracellular CDCP1 between high- and low-risk prostate cancer **(A)** Detection of CDCP1 in urine-EPS samples. Western blot analysis of urine-EPS samples from low-risk, intermediate-risk, and high-risk PCa were performed with a goat antibody against the extracellular domain (aa 33–666) of CDCP1 (AF2666). **(B)** Scheme for immune-enriched quantitative mass spectrometry analysis of CDCP1 proteins from urine-EPS. CDCP1 was immunoprecipitated with anti-CDCP1 (AF2666) from urine-EPS as described. Goat IgG was included as an isotype control. Eluents of the purified CDCP1 proteins were subjected to SDS-PAGE. Gel bands were cut into 15 equally spaced pieces (25–150 kDa) and subjected to LC-MS/MS analysis. **(C)** Identification of peptides spanning extracellular domains of CDCP1 detected by LC-MS/MS. **(D)** Representative MS/MS spectrum of tryptic peptide TFIWDVK of sCDCP1 digestion. **(E)** Quantitative precursor MS results display simultaneous identification and quantitation results for the 6 unique peptides from CDCP1. The left panel shows the relative peak area differences for the combined targeted peptides. The right panel shows the extracted ion chromatogram and integrated peak area of primary ions per targeted peptide.

A number of integral cell surface proteins, such as c-Met, CD44 and EGFR are also produced as soluble molecules with potential diagnostic utility [33–35]. We utilized antibodies specific for the CDCP1 ectodomain to analyze patient-derived urine. As shown in Figure 7A, analysis of urine samples with antibody targeted to the ectodomain detected several CDCP1 species with apparent molecular weights ranging from 110 kD to 25 kD. This is the first report of circulating CDCP1 detected in prostate cancer patients.

Having determined that an extracellular form of CDCP1 can be detected in urine, we established an immuno-MS workflow for quantitative detection of CDCP1 (Figure 7B). For this purpose we used the ectodomain-targeted antibody to immunoprecipitate CDCP1 directly from urine-EPS. Due to the low abundance of CDCP1, we pooled and concentrated samples from high-risk and low-risk prostate cancer patients. We then performed immunoprecipitation on normalized pooled samples as described. The immunoprecipitate was subsequently fractionated by SDS-PAGE and individual gel regions excised and subjected to LC-MS/MS analysis. We detected CDCP1 isoforms from gel bands suggesting multiple forms ranging from 45–85 kDa derived from low-risk and high-risk pools (Figure 7C and 7D). We did not detect CDCP1 following immunoprecipitation with antibody isotypes controls (data not shown).

We next performed label free quantitation of the immunoprecipitated CDCP1 in each patient risk group by precursor ion quantitation. This method sums all detected CDCP1 ions for a higher degree of confidence in the quantitative analysis. We observed that CDCP1 was present at significantly higher levels in the high-risk compared to low-risk patients (Figure 7E). These data indicate that extracellular forms of CDCP1, detectable in clinical fluids, have the potential to discriminate between prostate cancer risk groups.

## DISCUSSION

CDCP1 has emerged as an important tumor-regulating membrane bound protein. Although the mechanism of action of CDCP1 in facilitation of metastasis still avoids a consensus, blocking the proteolytic release of soluble ectodomains and coincident formation of the 70 kDa membrane-bound fragment inhibits early metastatic events [30]. Thus, the post-translational processing of CDCP1 appears to play an integral role in the regulation of this important protein. So, we postulated that differential glycosylation, perhaps via altered proteolysis, regulates CDCP1 expression and its role in cancer progression. Our current results support this hypothesis in several aspects. First, we demonstrated that CDCP1 glycosylation is extensive and variable comprised of complex type N-glycans and sialic acid, providing structural diversity needed for regulation of function. Second, inhibition of N-linked glycosylation altered the relative expression of HMW and LMW forms of CDCP1, induced the expression of degraded species and prevented cell surface presentation. Thus, N-glycosylation of CDCP1 impacts proteolytic processing and subcellular localization of CDCP1, which in turn contributes to protein expression and stability. Furthermore, we uncovered differential glycan structure of HMW-CDCP1 between “normal” and “metastatic” cellular phenotypes. Therefore, we postulate that the glycosylation of CDCP1 serves as an extracellular signal, together with intracellular phosphorylation, that modulates cell adhesion, anchorage-independent growth and metastasis.

Functionally, CDCP1 likely provides a basal activity that enforces anchorage-dependent growth since ablation of CDCP1 expression promotes tumor metastasis [36,37]. However, in model systems in which CDCP1 expression is dysregulated it provides clear tumor promoter activities [17–21, 28, 30, 31]. The “switch” in this process appears to be extracellular cleavage of CDCP1 allowing for Src-mediated signaling [10] and direct interaction with integrin [31] to drive migration and invasion. Thus, CDCP1 displays a profound structural/functional shift concomitant with tumor progression. There is precedence that compartmentalization of CDCP1 can regulate this functional shift. Specifically, CDCP1 localization to membrane lipid rafts was shown to be a prerequisite to invadopodia-mediated cell invasion [17], and cell migration in an ovarion cancer model required EGF-induced relocalization of CDCP1 to the cell surface [38]. We propose that glycosylation of CDCP1 dictates cell surface presentation and proteolytic processing to drive the functional switch between tumor suppressor and tumor promoter activities.

In light of its role in cancer progression, CDCP1 tissue expression has been examined as a potential cancer biomarker with conflicting results. For instance, increased CDCP1 expression correlated with poor prognosis in lung and colorectal [39, 40] but better outcomes in esophageal cancer [41]. In our study, we evaluated CDCP1 expression in both frozen and FFPE tissues. Although examination of CDCP1 expression in frozen prostate tissues by immunofluorescence microscopy revealed significant differences in subcelluar distribution between cancer and adjacent normal, these results were not recapitulated in our survey of FFPE tissue. In FFPE tissues, the expression pattern in cancerous and normal glands was heterogeneous, likely the result of antigen structural changes during FFPE tissue processing. Our assessment of CDCP1 expression in an FFPE tissue array demonstrated a modest decrease in tumor compared to normal, consistent with an earlier report [24]. Given current affinity reagents, CDCP1 expression in FFPE tissues does not appear to provide diagnostic value for prostate cancer.

The proteolytic cleavage of the CDCP1 extodomain yields a soluble 65 kDa extracellular polypeptide when examined in DU145 cells [8, 25]. In the present study we analyzed both soluble and vesicle-associated extracellular species of CDCP1. We identified membrane bound HMW/LMW forms of CDCP1 and demonstrated that a Type I transmembrane orientation is recapitulated in extracellular vesicles produced by PCa cells. In addition, we observed a 65 kDa and novel 110 kDa soluble form that was enriched in serum free culture medium depleted of microvesicles. A novel membrane-bound 25 kDa species containing the cytoplasmic domain was observed enriched in extracellular microvesicles. The presence of a 110 kDa extracellular protein and a 25 kDa intracellular protein suggested an additional site of proteolytic cleavage of the CDCP1 ectodomain proximal to the extracellular membrane border. Our study provides evidence of novel processing ofextracellular CDCP1 that results in both soluble and vesicle-associated species.

Immunoprecipitation of CDCP1 followed by sensitive mass spectrometry, was able to confirm the existence of extracellular forms of CDCP1 in urine of PCa patients. Moreover, using quantitative mass spectrometry we demonstrated that increased levels of CDCP1 correlated with aggressive disease. Although follow-up analysis with improved assay sensitivity allowing for analysis of individual samples is needed, the results suggest that extracellular species of CDCP1 may serve to discriminate aggressive from indolent disease.

In summary, our data reveal that N-glycosylation is a prerequisite to processing and membrane surface presentation of CDCP1. Increased sialylation and cell surface presentation of CDCP1 signals a transition between epithelial and tumor cell expression. These and other phenotypic changes in CDCP1 are faithfully conserved in extracellular vesicles derived from prostate cancer cells. We propose that dysregulated processing and expression of extracellular CDCP1 reflect the tumor microenvironment and a full structural characterizationis warranted to determine if circulating CDCP1 can provide a diagnostic/prognostic tool for management of prostate cancer.

## MATERIALS AND METHODS

### Patient samples

Patient samples were collected from consented men following Institutional Review Board approved protocols. We stratified patients to D'Amico's risk categories; low-risk (serum PSA < 10 ng/mL, Gleason score < 7, or clinical stage < T2b), intermediate-risk (serum PSA between 10 ng/mL and 20 ng/mL, or Gleason score equal to 7, or clinical stage T2b) or high-risk (serum PSA > 20 ng/mL, or Gleason score 8 to 10, or clinical stage T2c-3a). Urine-EPS samples were collected as described [42]. Urine-EPS specimen pools comprised 10 samples (12 ml each) for a total volume of 120 ml. The clinicopathological characteristics of urine-EPS samples are summarized in Table S1. Each pool was concentrated 200X by ultrafiltration.

### Cell culture and reagents

Human prostate epithelial cell lines RWPE-1 and PZ-HPV-7, RWPE-1 sublines (WPE1-NB14, WPE1-NB26 and WPE1-NB26/65), VCaP, LNCaP, DU145, 22RV1, HEK-293T, and MCF-7 were obtained from American Type Culture Collection. PC3 sublines PC3-N2 and PC3-ML2 [43] were provided by Mark E. Stearns (Drexel University, PA). The LNCaP sublines C4, C4–2 and C4–2B were developed by Leland W. Chung [44] and provided to us by ViroMed Labs. ARCaP cells and subclones were purchased from Novicure Biotechnology (Birmingham, AL). PacMetUT1 was developed by Dean A. Troyer [45]. Human prostate epithelial cell line HPrEC was purchased from Lifeline Cell Technology (Frederick, MD). CDCP1 antibodies used were: mouse monoclonal mAb 41–2 [8]provided by James P. Quigley (The Scripps Research Institute, CA); rabbit polyclonal CS4115 (Cell Signaling, Danvers, MA); mouse monoclonal mAB309137 and goat polyclonal AF2666 (R&D Systems, Minneapolis, MN). Murine monoclonal anti-β-actin was from BD PharMingen (San Diego, CA) and rabbit polyclonal anti-GAPDH was from Santa Cruz Biotechnology (Santa Cruz, CA). IRDye 700 and IRDye 800 conjugated secondary antibodies were from Li-COR Bioscences (Lincoln, PA). Alexa Fluor 488 conjugated secondary antibodies were from Invitrogen (Carlsbad, CA). PNGase F, neuraminidase, and Endoglycosidase H were from New England Biolabs (Beverly, MA). High capacity streptavidin agarose resin and protein A/G PLUS-agarose were from Thermo Scientific (Rockford, IL) and Santa Cruz Biotechnology (Santa Cruz, CA), respectively.

### Statement on authentication of cell lines

Phenotypic verification was conducted upon receipt of cell lines and no genetic authentication was performed. All cultures were used at passage 2–8. The morphology and expression of EMT markers (E-cadherin, N-cadherin, vimentin) was confirmed and cytokeratin 18 expression measured to verify epithelial origin. The expression of adhesion surface molecules (CDCP1, basigin, EGFR, Integrin) was determined.

### Immuno- and affinity blotting

Whole cell lysates were collected in lysis buffer containing 1x protease inhibitor cocktail. Protein concentration was measured by the BCA protein assay (Thermo Scientific, Rockford, IL), separated by electrophoresis through 4–12% or 7.5% SDS-PAGE and then transferred to Immobilon-FL PDVF membranes (Millipore, Billerica, MA). Membranes were blocked in LiCor blocking buffer (LiCor, Lincoln, NE) diluted with PBS (1:1), then incubated with primary antibodies overnight at 4°C. Following extensive washing, membranes were incubated with species-specific IRDye700 or 800-conjugated secondary antibodies (1:15,000) for 1 h at room temperature, and visualized with a LiCor Odyssey infrared imager (LiCor, Lincoln, NE). Consistent protein loading was determined by reprobing membranes with anti-actin or anti-GAPDH antibodies. To probe with lectins, the protein blot was blocked with protein-free blocking buffer (PFB, Thermo Scientific, Rockford, IL) at room temperature for 1 h, and then probed overnight at 4°C with 1 μg/ml of biotinylated lectins (Vector Laboratories, Burlingame, CA) in lectin-binding buffer (50 mM Tris, pH 7.5, 0.15 M NaCl, 0.1 mM CaCl_2_). The filters were then washed with TBST (TBS with 0.05% Tween-20), incubated with IRDye 800-conjugated streptavidin in PFB for 1 h and then washed with TBST. The blots were imaged on the LiCor Odyssey system.

### Cell surface biotinylation

N2 and ML2 cells were cultured in the absence or presence of tunicamycin (1 μg/ml) for 24 h. The cells were then washed three times with ice-cold PBS. Cell surface proteins were subsequently biotinylated with 0.4 mg/ml Sulfo-NHS-LC-biotin (Pierce, Rockford, IL) in ice-cold PBS for 45 min. Nonbiotinylated control cells were washed with ice-cold PBS. The reaction was quenched by washing cells three times with TBS (50 mM Tris, 150 Mm NaCl, PH 7.4). Cells were then lysed in RIPA buffer (0.5% sodium deoxycholate, 1% Nonidet-P 40, 0.1% SDS, 2mM EDTA in Tris solution) containing protease inhibitors. The cell lysates were centrifuged at 14,000 *g* for 15 min to remove insoluble materials. The protein concentration was tested by BCA assay. To bind the biotinylated proteins, high capacity streptavidin-agarose beads (Thermo Scientific, Rockford, IL) were blocked with protein-free blocking buffer at 4°C overnight followed by TBS wash three times. 50 μl pretreated beads were added to each 1 mg supernatant lysate and incubated for overnight at 4°C. The beads were settled by centrifugation, and the supernatants (unbound fractions) were saved. The beads were then washed five times with RIPA buffer, and bound proteins were eluted by the addition of Laemmli buffer.

### Characterization of protein glycosylation

For glycosidase digestion, 20 μg cell lysates were mixed with 1,000 NEB units PNGase F, 1,000 NEB units Endo H, or 50 NEB units neuraminidase as indicated. For glycosylation inhibition assays, sub-confluent cells were washed twice with PBS and cultivated for 24 h in fresh culture media in the presence of 5 μg/ml tunicamycin or 1 μg/ml swainsonine. Cells were washed twice and lysed in M-PER buffer containing protease inhibitor. The lysates were centrifuged at 14,000 g for 15 min and supernatant collected.

### Assessment of overall sialylation

N2 and ML2 cells were treated with 25 nMazido-modified sugars, tetraacetylated N-azidoacetyl-D- mannosamine (AC_4_ManNAz, Invitrogen, Carlsbad, CA), and normal control sugars, N-acetyl-D-mannosamine (ManNAc) for 24 h. Cells were lysed in M-PER lysis buffer with protease inhibitor. After CDCP1 was immunoprecipitated from the indicated amount of cell lystes, click reaction was performed with biotinylated alkyne capture reagent (0.1 mM alkyne biotin, 0.1 mM Tris-triazoleamine catalyst, 1 mMCuSO_4_, 2 mM sodium ascorbate) on eluted protein at room temperature for 1 h. Unreactive reagents was removed by chloroform and methanol precipitation followed by solublizing protein in NuPAGE sample loading buffer (Invitrogen, Carlsbad, CA) and analyzed by Western blot with IRDye 800 conjugated streptavidin.

### Immunofluorescence staining

Cells were seeded onto 6-well plates containing glass coverslips and cultured in growth medium to 80% confluence, washed with ice-cold PBS and fixed in 4% paraformaldehyde for 15 min at room temperature. For intracellular staining, the cells were permeabilized with 0.5% Triton X-100 in PBS for 10 min at room temperature. Fixed cells were washed twice with PBS and blocked with 3% BSA in PBS for 1 h at room temperature. Cells were then incubated with primary antibody in blocking buffer overnight at 4°C, washed three times and incubated with the appropriate conjugated secondary antibodies (2 μg/ml, Invitrogen, Carlsbad, CA) in the blocking buffer for 1 h at room temperature. Nuclei were counter-stained with TO-PRO3, coverslips mounted to slides with VectorShield (Vector Labs, Burlingame, CA), and sealed with nail polish. Cells stained with normal goat or rabbit IgG were included as negative controls.

For tissue staining, Optimal Cutting Temperature reagent-embedded frozen PCa tissues were obtained from the Leroy T. Canoles Jr. Cancer Research Center biorepository. Cryosectioning was done on a Microm HM 505E cryostat at −20°C. A serial cryosection (6 μm) was stained with H&E as a guide and analyzed by a pathologist to determine tissue morphology. Tissue sections were fixed in 4% paraformaldehyde in PBS for 15 min, permeabilized with 0.5% Triton X-100 in PBS for 10 minutes, blocked with 3% BSA in PBS for 1 h and incubated overnight at 4°C with primary antibody. Excess primary antibody was removed and the cells incubated with the appropriate conjugated secondary antibody in the blocking buffer for 1 h at room temperature. Nuclei were counter-stained with propidium iodide. An adjacent section stained with mouse normal IgG was used as the negative control. Imaging was done on an Olympus BX51 fluorescent microscope or a Zeiss LSM510 META confocal imaging system.

### Preparation of extracellular vesicles

Cells were grown to 80% confluence, rinsed and cultured in serum-free medium for 48 h. Culture media was harvested and vesicles enriched by ultracentrifugation as described [46]. Briefly, the media were subjected to serial centrifugation to remove cells (500x g for 10 min), dead cells (2,000x g for 20 min), and cell debris (20,000x g for 30 min). Vesicles were then collected by ultracentrifugation at 100,000x g for 90 min at 4°C using a SW28 rotor in an Optima X-100K ultracentrifuge (Beckman Coulter), washed with a large volume of ice-cold PBS, and concentrated by ultracentrifugation at 100,000x g for 90 min at 4°C in a SW40 rotor. Vesicle pellets were resuspended in PBS or lysis buffer and frozen at −80°C. The quantity of Vesicles was determined by Bradford protein assay (Thermo Scientific, Rockford, IL).

### Flow cytometry analysis

For cell surface staining, subconfluent cells were harvested from the culture dishes by non-enzyme dissociation buffer (Invitrogen, Carlsbad, CA), washed with 2% FBS/PBS, and incubated with primary antibody AF2666 (5 μg/ml) for 1 h at 4°C. Cells were washed twice and incubated with Alexa Fluor 488-conjugated donkey anti-goat antibody (2 μg/ml, Invitrogen, Carlsbad, CA) for 30 min at 4°C. Cells were then treated with propidium iodide (1 μg/ml) in 500 μl 2% FBS/PBS for 20 min at 4°C. Cells stained with goat normal IgG were used as the isotype controls. Flow cytometry of isolated extracellular vesicles was performed as described [46]. Briefly, vesicle-coated beads were prepared by incubating purified extracellular vesicles (5 μg protein) with 4 μm aldehyde/sulfate latex beads (5 μl of 4% w/v suspension, Invitrogen, Carlsbad, CA) in PBS for overnight at 4°C under constant agitation. The reaction was stopped by addition of 1M glycine, followed by three washes in FACS buffer (2% FBS/PBS). Vesicle-coated beads were then incubated with goat polyclonal anti-CDCP1 AF2666 antibody (5 μg/ml) or rabbit polyclonal CS4115 (5 μg/ml), anti-CD9 (5 μg/ml, clone M-L13, BD Biosciences, San Jose, CA) and anti-CD63 (5 μg/ml, clone H5C6, BD Biosciences, San Jose, CA) or isotype control (5 μg/ml, Santa Cruz Biotechnology, Santa Cruz, CA) followed by Alex Fluor 488-conjugated secondary antibodies (4 μg/ml, Invitrogen, Carlsbad, CA). Flow cytometry was performed on a FACSAria instrument (BD Biosciences), and data were analyzed using Flowjo software (Tree Star Inc).

### Immuno-mass spectrometry analysis of circulating CDCP1

1.2 mg total protein of concentrated Urine-EPS pools was pre-cleared with goat IgG, incubated with 5 μg goat anti-CDCP1 (AF2666) and immunoprecipitated. Bound proteins were eluted and subjected to SDS-PAGE. Gel regions were excised, destained and washed with a series of three washing buffers (50 mM ammonium bicarbonate, 50% acetonitrile and 80% acetonitrile). Proteins were reduced, alkylated and digested as previously described [47]. Dried samples were dissolved with 20 μl of 0.1% formic acid/water. 2 μl of each sample was analyzed by LC/ESI-MS/MS using a Q-Exactive (Thermo Fisher Scientific, Bremen, Germany) mass spectrometer with an Easy NanoLC-1000 system using data dependent acquisition with dynamic exclusion (DE = 1). MS acquisition parameters used have been described elsewhere [48]. Pinpoint (version 1.1, Thermo Scientific) was employed to determine optimal peptide targets. Retention time and accurate m/z for each targeted peptide was used in the acquisition method for scheduled MS/MS. Six of the identified peptides were selected and exported as a retention time-dependent inclusion list to build the acquisition method. Quantitation analysis was performed in triplicate using an automated fashion using a 5 ppm window for extracted ion chromatograms.

### Construction and immunohistochemical analysis of tissue microarray

Formalin fixed paraffin embedded tissue blocks were obtained from men undergoing radical prostatectomy between 1990 and 2006 at Sentara Norfolk General Hospital (Norfolk, VA) under an IRB-approved protocol for development of Retrospective Prostate Tissue Microarrys. The clinicopathological characteristics of the patients are summarized in table 1. Three TMAs consisting of 100 cases of primary prostate cancer with matched benign tissues were generated. After evaluation by a pathologist, three representative cores (1.0 mm in diameter) and one core from matched normal tissue were taken from a randomly selected tumor block reflecting the Gleason grade of the pathological diagnosis from each patient and arranged in tissue microarray blocks using TMA Arrayer (Pathology Devices, Westminster, MD). For internal controls, each TMA block contained normal prostate, kidney, liver, tonsil and colon tissue. 4 μm sections from TMA blocks were deparaffinized and rehydrated in xylene and graded ethanol baths. The antigen was retrieved in 10 mM sodium citrate (pH 6.0) in boiling water for 20 min. Slides were then blocked with 10% normal horse serum in 1% BSA/PBS at room temperature for 1 h and incubated overnight at 4°C with primary mouse anti-CDCP1 antibody mAb309137 (0.5 μg/ml) or mouse isotype antibody (negative control). The next day slides were blocked with 3% H_2_O_2_ and avidin/biotin blocking kit (Vector Lab). Secondary staining was performed with biotinylated horse anti-mouse antibodies (50 μg/ml, Vector Lab) and visualized with Vectastain ABC kit (Vector Lab) and 3, 3′-diaminobenzidine (DAB)- H_2_O_2_ Substrate (BD Biosciences). Slides were counterstained with haematoxylin, dehydrated with ethanol/xylene and mounted. Slides were viewed and imaged under bright-field mode on a Zeiss AxioImager Z1 microscope fitted with an AxioCamMRc digital camera (Carl Zeiss, Thornwood, NY). Semi-quantitative assessment of immunoreactivity was performed by a pathologist in a blinded fashion. The staining intensity was graded as weak (1 +), moderate (2 +) or strong (3 +). The total IHC score was calculated by multiplying the percentage of positive cells (0 – 100%) and staining intensity (1 + to 3 +) so that CDCP1 IHC score ranged from 0–300.

**Table 1:** Clinicopathological Features of Patient Samples Used to Make EPS-urine Pools

Sample #	StageT	GS	PSA (ng/ml)	Cores	Pos	%Pos
**Low-risk group**						
1	T1c	3 + 3	3.37	12	4	33.33
2	T1c	3 + 3	4.35	12	2	16.67
3	T1c	3 + 3	7.29	12	1	8.33
4	T1c	3 + 3	4.00	12	4	33.33
5	T1c	3 + 3	5.10	12	2	16.67
6	T1c	3 + 3	3.40	12	6	50.00
7	T1c	3 + 3	3.90	12	3	25.00
8	T1c	3 + 3	4.80	12	5	41.67
9	T1c	3 + 3	4.08	12	1	8.33
10	T1c	3 + 3	4.00	12	2	16.67
**Intermediate-risk group**						
1	T1c	4 + 3	5.30	12	5	41.67
2	T2a	4 + 3	3.58	12	5	41.67
3	T1c	4 + 3	15.80	12	8	66.67
4	T1c	4 + 3	5.38	12	6	50.00
5	T1c	4 + 3	8.10	12	4	33.33
6	T1c	4 + 3	5.10	12	2	16.67
7	T2	4 + 3	8.41	14	8	57.14
8	T2b	4 + 3	4.30	12	12	100.00
9	T1c	4 + 3	10.90	12	5	41.67
10	T1c	4 + 3	4.70	12	3	25.00
**High-risk group**						
1	T2a	5 + 4	0.53	12	11	91.67
2	T2a	4 + 4	32.49	13	5	38.46
3	T1c	5 + 4	80.90	12	6	50.00
4	T1c	4 + 4	9.05	12	10	83.33
5	T2c	4 + 5	27.90	12	9	75.00
6	T2a	4 + 5	9.38	12	6	50.00
7	Unkown	4 + 4	52.99			
8	T2a	4 + 5	8.30	12	12	100.00
9	T1c	4 + 4	6.28	12	3	25.00
10	T2c	4 + 5	2.70	15	5	41.67

Abbreviations: PSA, prostate-specific antigen; Pos, positive cores; GS, Gleason score.

## SUPPLEMENTARY MATERIALS FIGURES AND TABLE


