# Supplementary material for: Dysregulated expression of cell surface glycoprotein CDCP1 in prostate cancer
Source: Oncotarget. 2015 Oct 20;6(41):43743–58. doi: 10.18632/oncotarget.6193 (PMC4791263; doi:10.18632/oncotarget.6193)
Supplement: Supplementary file 1 [file oncotarget-06-43743-s001.pdf]

Dysregulated expression of cell surface glycoprotein CDCP1 in prostate cancer

Supplementary Materials

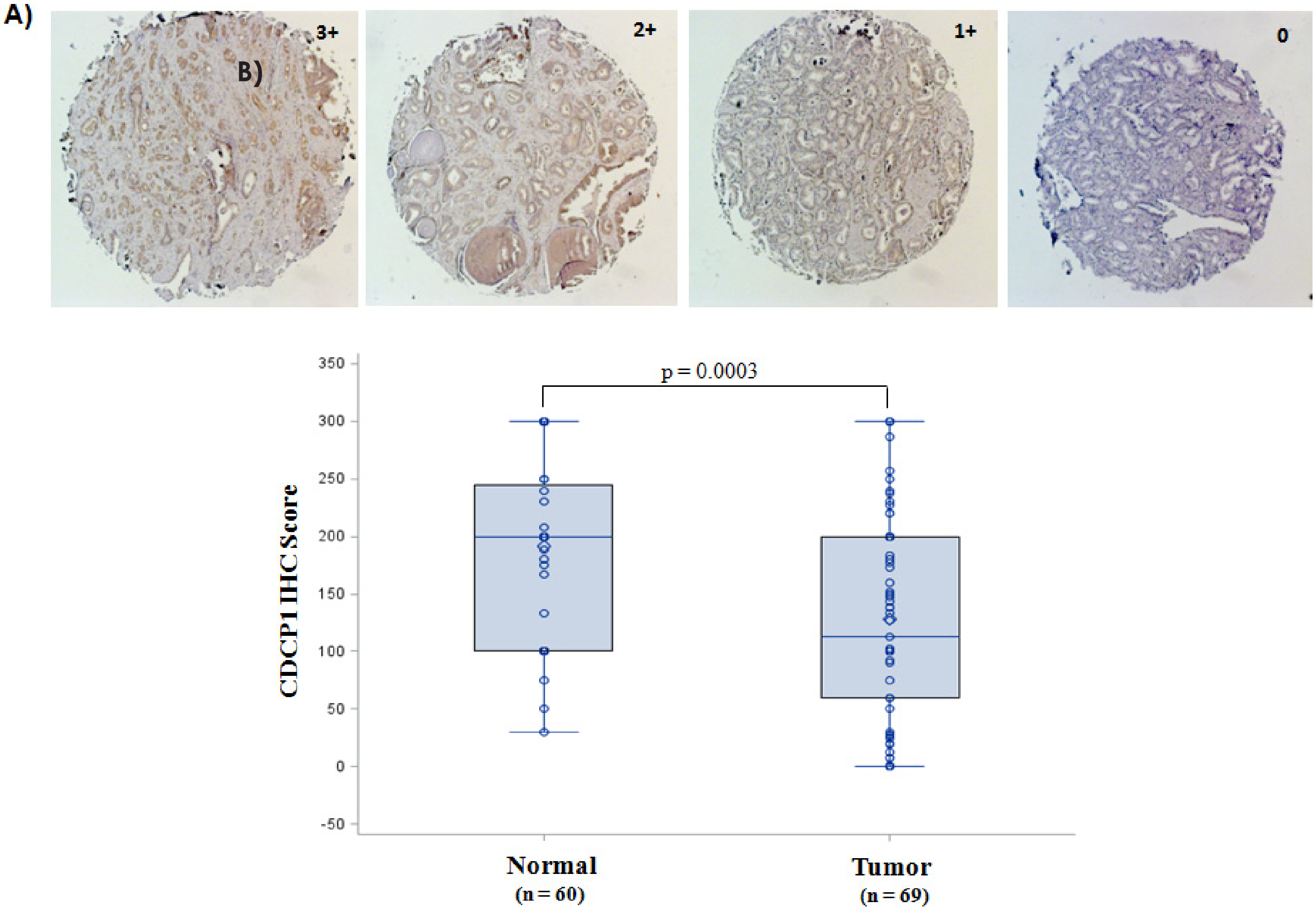

Supplementary Figure 1: Immunohistochemical staining of CDCP1 in FFPE sections of human prostate cancer. (**A**) Examples of CDCP1 immunostaining in human PCa tissues. (**B**) Distribution of CDCP1 IHC Scores. Box plot shows the relation between CDCP1 expression in the noncancerous (N) and cancerous (T) tissues. The line within the box plot corresponds to the median value, the box length to the score range, and the lines emanating from the box (whiskers) to the variations. Statistical analyses were done by signed rank test using SAS 9.3 software.

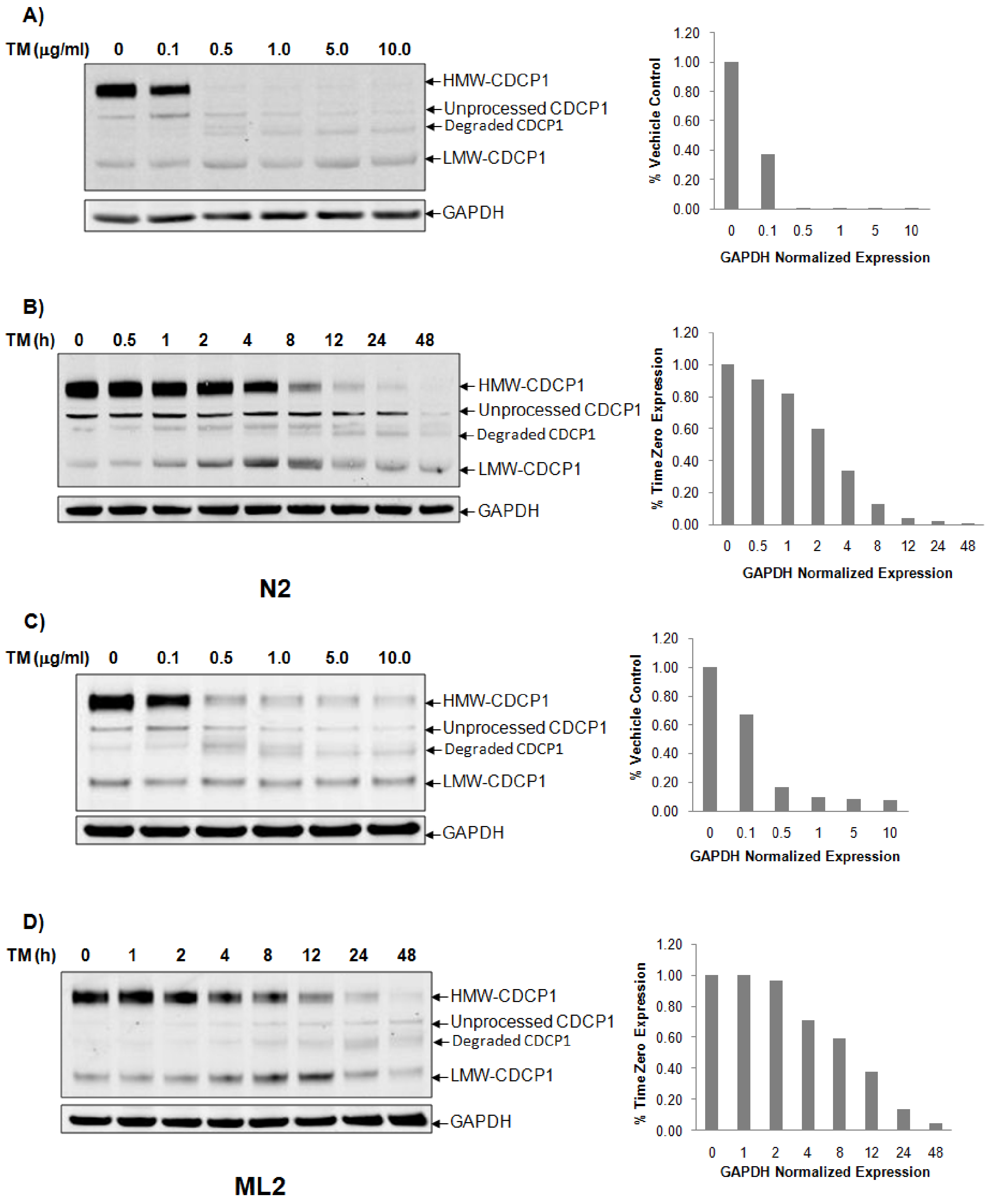

Supplementary Figure 2: Shown is a dose response for the effect of tunicamycin (TM) on CDCP1 expression in PC3-N2 (A) and PC3-ML2 (C). Cells were incubated for 24 h with the indicated doses of tunicamycin. A time course for CDCP1 expression in PC3-N2 (**B**) and PC3-ML2 (**D**) with the concentration of tunicamycin held constant. Cells were incubated with 1 µg/ml tunicamycin for the indicated time. CDCP1 expression was examined by western blot analysis using anti-CDCP1 (CS4115 antibody). The normalized expression of CDCP1 is presented for each condition (right panels).

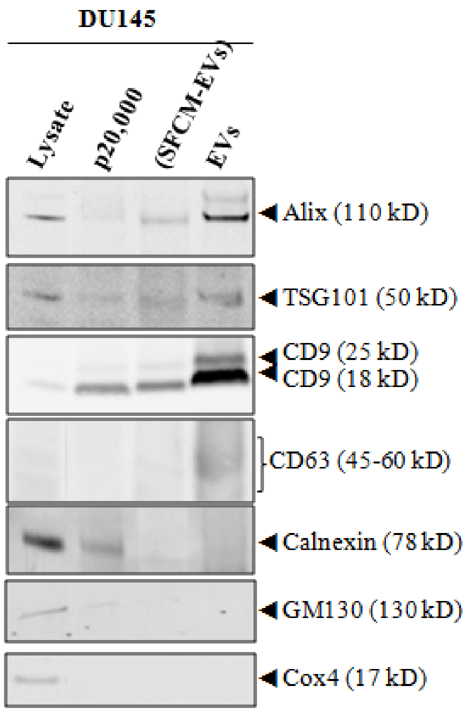

Supplementary Figure 3: Characterization of EVs Derived from DU145 Cell Line. Western blot analysis of common exosome markers (Alix, TSG101, CD9 and CD63) and cell organelle markers (Calnexin for ER, GM130 for Golgi, and Cox4 for mitochondria) in whole cell lysates, large vesicles precleared with 20,000 g (p20,000), SFCM depleted of extracellular vesicles (SFCM-EVs), and extracellular vesicle (EVs) fractions isolated from DU145 cells. All fractions were loaded with 20 µg of protein except 40 µg for (SFCM-EVs) fraction.

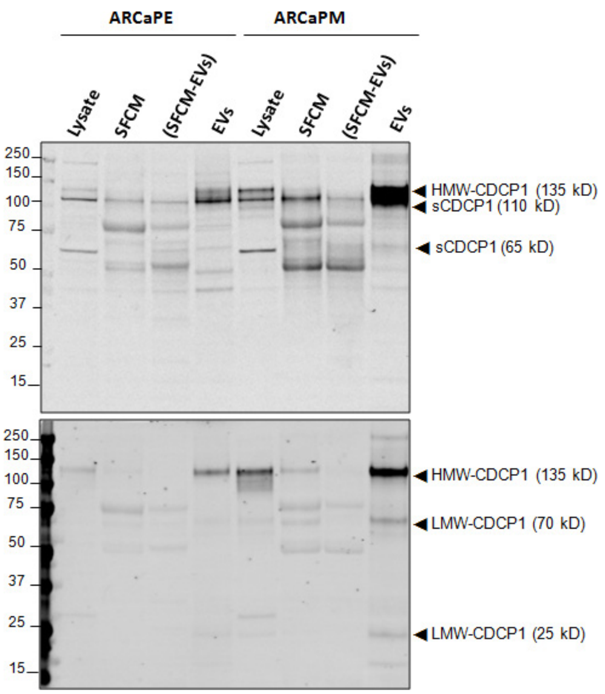

Supplementary Figure 4: Analysis of Extracellular CDCP1 Derived from ARCaP Cell Lines. Whole cell lysates, serum-free conditioned medium (SFCM), SFCM depleted of extracellular vesicles (SFCM-EVs) and extracellular vesicle (EVs) fractions derived from ARCaPE and ARCaPM cells were analyzed as indicated. The same transfer filter was reacted with anti-CDCP1 specific to the extracellular domain (mAB309137, upper panel) and to the intracellular domain (CS4115, lower panel). 20 µg of protein was loaded in lysate and EVs fraction while 40 µg was loaded in SFCM and SFCM-EVs fractions.

Supplemental Table 1: Clinicopathological Features and CDCP1 Expression in Patients with PCa

Features	Total	Low	High	*p*
**Prostate cancer**				
n (%)	69 (100.0)	35 (50.7)	34 (49.3)	
**Age at surgery (years), mean (range)**	62 (50–73)	60 (52–73)	62.5 (50–72)	0.26
**Pretreatment PSA (ng/ml), mean (range)**	7.4 (0.5–23.7)	7.5 (0.5–20.9)	7.0 (3.5–23.7)	0.71
**Clinical Stage, *n* (%)**				
T1	44 (64.7)	24 (70.6)	20 (58.8)	
T2	23 (33.8)	9 (26.5)	14 (41.2)	
T3	1 (4.7)	1 (2.9)	0 (0.0)	0.23
**Pathologic tumor stage, *n* (%)**				
pT2	29 (42.0)	14 (40.0)	15 (44.1)	
pT3	36 (52.2)	18 (51.4)	18 (52.9)	
pT4	4 (5.8)	3 (8.6)	1 (3.0)	0.89
**Prostectomy Gleason grade, *n* (%)**				
≤ 6	32 (46.4)	16 (45.7)	16 (47.1)	
7	28 (40.6)	12 (34.3)	16 (47.1)	
≥ 8	9 (13.0)	7 (20.0)	2 (6.0)	0.22
**Extracapsular extension (ECE), *n* (%)**				
No	39 (59.1%)	20(58.8)	19 (59.4)	
Yes	27 (40.7%)	14 (41.2)	13 (40.6)	
**Seminal vesicle invasion (SVI), *n* (%)**				
No	53 (77.9%)	27 (77.1)	26 (78.8)	
Yes	15 (22.1%)	8 (22.9)	7 (21.2)	0.87
**Surgical margin status (SMS), *n* (%)**				
No	41 (62.1%)	20 (60.6)	21 (639.4)	
Yes	25 (37.9%)	13 (39.4)	12 (36.6)	0.79
**Pathological percentage cancer (%), mean (range)**	15 (1–95)	20 (1–90)	11.5 (1–95)	0.04
**Biochemical recurrence (BCR), *n* (%)**				
No	18 (26.1%)	8 (22.9)	10 (29.4)	
Yes	51 (53.9%)	27 (77.1)	24 (70.6)	0.53

*CDCP1 IHC score ≤ 113 was considered as "low CDCP1 expression" and score > 113 as "high CDCP1 expression". 2. Statistical analyses were performed using SAS 9.3 software. Chi-square test was used for categorical data while Wilcoxon Mann Whitney test and logistic regression were applied for quantitative data
